# How does Frailty Impact Peri‐Operative and Speech Recognition Outcomes for Cochlear Implants in Veterans?

**DOI:** 10.1002/ohn.70140

**Published:** 2026-02-01

**Authors:** Kaitlyn A. Brooks, Benjamin D. Lovin, Alex D. Sweeney, Angela S. Peng, Nathan R. Lindquist

**Affiliations:** ^1^ Bobby R. Alford Department of Otolaryngology–Head and Neck Surgery Baylor College of Medicine Houston Texas USA; ^2^ Department of Otolaryngology–Head and Neck Surgery University of Virginia Charlottesville Virginia USA; ^3^ Otolaryngology‐Head and Neck Surgery Section Michael E. DeBakey Veterans Affairs Medical Center Houston Texas USA

**Keywords:** admission, cochlear implantation, frailty, postoperative dizziness, veterans

## Abstract

**Objective:**

To investigate the association of frailty with post‐cochlear implant (CI) admission, morbidity, and CI‐aided word recognition outcomes in veterans.

**Study Design:**

Retrospective cohort.

**Setting:**

Single‐institution tertiary care Veterans Health Administration (VHA) hospital.

**Methods:**

Veterans who underwent cochlear implantation between 1998 to 2024 were included. The Modified 5‐Item Frailty Index (mFI‐5) score was used to characterize preoperative frailty. Outcomes of interest were admission from post‐anesthesia care unit (PACU), relative risk (RR) of admission among frail patients, and relationship between frailty and post‐CI word recognition score (WRS). Ordinal data were analyzed via logistic regression and Chi square tests; a multivariate linear regression was used to assess nominal data. Significance was set at *P* < .05.

**Results:**

Ninety‐one patients (median age 71 years, range 35‐92 years) resulted in 41 (39.4%) admissions out of 104 surgical encounters. Admission rate initially increased from 2016 to 2020 (48.9%), then decreased (29%) in 2021. Forty‐two (46%) patients met criteria as being frail, while 26 (28.6%) were prefrail. While frail patients were more readily admitted (43% vs 36%; RR 1.15) and had higher rates of dizziness (20% vs 15%; RR 1.23) when compared to the combined cohort of nonfrail and prefrail patients, these relationships were not significant. Preoperative frailty was significantly negatively associated with post‐CI WRS at 6‐ and 12‐month post‐CI in a multivariate analysis including patient age (*P* < .05).

**Conclusion:**

The prevalence of frailty is high among veterans undergoing cochlear implantation. Patient frailty is significantly associated with post‐CI speech recognition but did not appear to impact likelihood of admission or complications.

Veterans suffer from high rates of hearing loss,^1^, ^2^, ^3^, ^4^ and undertreated hearing loss is associated with social isolation, risk of anxiety and depression, and dementia.^5^, ^6^ For patients with moderate‐to‐severe hearing loss and limited benefit from traditional amplification, cochlear implantation (CI) is the gold standard for hearing rehabilitation, though patients must be able to safely undergo the surgical procedure and participate in post‐operative aural rehabilitation.^7^, ^8^ Increasingly, the analysis of CI performance has recognized quality of life benefits beyond pure speech recognition, even for those patients who are older or more frail.^9^, ^10^, ^11^, ^12^ Despite these results, the CI process remains purely elective and there may be hesitation when offering elective surgery to patients with significant medical comorbidities or extreme elderly age.^13^, ^14^, ^15^, ^16^ Notably, the veteran population has a higher rate of chronic conditions than non‐veteran patients, which inspires the question of whether cochlear implantation in this population may have unique considerations.^17^, ^18^, ^19^, ^20^, ^21^

Since 2010, the relationship between patient frailty and surgical outcomes has become better understood.^22^ Frailty is defined as a patient's physiologic reserve and ability to adapt to external stressors;^21^, ^23^ anecdotally, the potential for a slower recovery, an incomplete recovery, or a complicated recovery after surgery are concerning when offering elective procedures to frail patients. Preoperative frailty has been shown to portend mortality, postoperative complications, and length of stay independent of chronologic age in other surgical disciplines.^24^, ^25^, ^26^, ^27^, ^28^ Frailty, however, has been seldomly used to index the risk of postoperative complications^14^, ^29^ and post‐implantation outcomes among CI patients,^13^, ^30^, ^31^ despite the procedure's known ability to cause short‐term imbalance, which may increase risk for falls among frail patients.^31^, ^32^ Likewise, CI outcomes have been infrequently reported for veterans despite the high prevalence of hearing loss in this population and the readily‐available hearing rehabilitation afforded by the nationalized system of the Veterans Health Administration (VHA).^4^, ^33^, ^34^

The veteran population is known to have higher rates of chronic disease and frailty than civilian patients, which prompts the question of whether frailty in this patient population impacts outcomes of what is generally considered a well‐tolerated surgical procedure. Our objectives were to (1) assess severity and rates of frailty in veteran patients undergoing cochlear implantation, (2) determine if frailty affects peri‐operative admission and complications, and (3) investigate the association between pre‐operative frailty and word recognition abilities within the first‐year post‐implantation. We hypothesized that frailty would be associated with higher rates of postoperative admission and would be significantly associated with poorer word recognition scores.

## Methods

### Study Design & Setting

A retrospective cohort study was approved by the Michael E. DeBakey Veterans Affairs Medical Center Institutional Review Board (MEDVAMC 1773722, BCM H‐54164). The electronic medical record was queried to include all adult patients who received a CI for any diagnosis at our tertiary care referral VHA hospital between 1998 to 2024. Patients with concurrent labyrinthectomy and cochlear implantation were excluded from analysis due to the potential for confounding post‐operative dizziness/vertigo. Patients who did not have all desired demographic, audiological, and admission information readily available in the medical record were denoted as “unknown” for these variables yet still included in the study.

### Audiological Evaluation

Primary audiometric outcomes included monaurally obtained CI‐aided word recognition score (WRS), which included the open‐set AzBio sentence list and the consonant‐nucleus‐consonant (CNC) wordlist in quiet. Testing was performed by presenting the signal monoaurally to the ear to be implanted or to the implant being tested at 60 decibels hearing loss level. The signal was presented in a standard 0‐degree Azimuth configuration. This was in accordance with the minimum speech test battery for CI users. For English‐speaking patients, open‐set testing was performed via recorded media. For primarily Spanish‐speaking patients, a live version of the list was interpreted.

### Frailty Scores

The Modified 11‐Item Frailty Index is an 11‐factor frailty index that was developed using the American College of Surgeons NSQIP database by accumulating common factors seen among elderly patients.^35^ This system was further fine‐tuned to the Modified 5‐Item Frailty Index (mFI‐5), which is a concise and useful way to compare preoperative frailty and surgical outcomes.^36^, ^37^ Using the mFI‐5, we characterized patient frailty based on history of congestive heart failure (CHF), hypertension requiring oral anti‐hypertensive medication, type 2 diabetes mellitus (T2DM) requiring medication, chronic obstructive pulmonary disease (COPD), and functional independence within 30‐days of cochlear implantation. This was done by reviewing both primary care physician (PCP) and anesthesia preoperative documentation at the time of cochlear implantation to affirm the diagnosis was present at the time of CI. Functional independence was assessed per the Veterans Affairs Risk Analysis Index survey, which is a survey administered routinely in standardized VHA intake procedures which includes a patient‐reported item regarding functional status. Using the mFI‐5 allowed for a score to be generated per item of interest from 0 to 5 for each patient. Patients with mFI‐5 scores of 0 were nonfrail, patients with mFI‐5 scores of 1 were prefrail, and patients with mFI‐5 scores of 2 or greater were frail, consistent with prior literature.^14^, ^29^

### Complications

The electronic medical record was reviewed to determine complication rate within 90 days of implantation. Postoperative complications were divided as such: surgical complications (taste disturbance, post‐operative vertigo/dizziness, infection, implant extrusion, and facial nerve stimulation) and anesthesia complications (cardiac events such as myocardial infarction, arrhythmias, congestive heart failure exacerbation; allergic reactions; neurological events such as transient ischemic attack, stroke, or seizure; and urinary retention). Surgical complications that required operative or procedural intervention were labeled as major complications; all other surgical complications were characterized as minor complications.

### Primary and Secondary Outcomes

The primary outcomes were assessments of whether preoperative frailty influences (1) the need for overnight admission after CI in veterans and (2) if patient frailty affects CI outcomes within the first year after surgery in terms of monaurally obtained WRS. Secondary outcomes of interest included (1) admission rate over time, (2) patient frailty over time, and (3) whether post‐implantation dizziness/vertigo had a relationship with preoperative frailty and post‐CI admission.

### Statistical Considerations

The data obtained were found to follow a non‐normal distribution using the Shapiro‐Wilks test, prompting our decision to proceed with non‐parametric statistical analysis. Rate of admission and post‐operative dizziness were compared between all mFI‐5 scores using Chi‐Square test; an analysis between frail and prefrail patients (mFI‐5 ≤ 1) and frail patients (mFI‐5 ≥ 2) was performed with the Fischer Exact test. Continuous data were compared between mFI‐5 score groups using the Kruskal‐Wallis test. Appropriate test statistics are reported. Multivariate linear and logistic regression analyses were performed to assess relationships between frailty and other demographic variables with continuous and categorical outcomes, respectively, with 95% confidence intervals reported. *P *< 0.05 were set as significant. Statistical analysis was performed with SPSS Statistics (IBM Corporation) and GraphPad Prism (version 10.0.2; GraphPad Software).

## Results

Ninety‐one patients (104 ears) were included in our cohort with a median age of 71.0 years (IQR 63.9‐77.6, range 35.0‐92.5 years). Ninety (98.9%) patients were male. Table 1 shows cohort composition in terms of age at CI, admission rate, and vital status at time of data query by mFI‐5 score. Twenty‐three (25.3%) patients were nonfrail (mFI‐5 = 0), 26 (28.6%) patients were prefrail (mFI‐5 = 1), and 42 (46.1%) patients were frail (mFI‐5 ≥ 2).

**Table 1:** Frailty Levels and Rate of Admission for CI‐Related Encounters

mFI‐5 scores						
N(%) or Median (range)	0 Nonfrail	1 Prefrail	2 Frail	3 Frail	4 Frail	*P*‐value
No. of ears	26/104 (25%)	29/104 (28%)	37/104 (36%)	9/104 (9%)	3/104 (2%)	
Age (years)	63.6 (35.0‐82.4)	71.1 (54.1‐85)	72.1 (44.1‐92.5)	72.7 (57.9‐88.5)	76.9 (64.5‐78.6)	.004** (H = **15.1)** a
Pre‐CI AzBio^b^ (%)	17 (42‐0)	2 (0‐63)	14 (0‐58)	31 (0‐67)	40 (28‐48)	.11
Admission	10/26 (38%)	10/29 (34%)	15/37 (41%)	4/9 (44%)	2/3 (66%)	.88
LOA (days)	1 (1‐3)	1 (1‐2)	1 (1‐8)	1 (1‐8)	1 (1‐1)	.35
Vital status (death)	2/26 (7.8%)	4/29 (13.8%)	16/37 (43.2%)	3/9 (33.3%)	0/3 (0%)	.005** (*χ* = **13.1)** c
Time from CI to death (years)	6.3 (3.0‐9.6)	3.2 (1.1‐8.4)	4.7 (1.5‐9.7)	5.0 (4.9‐5.0)		.68

Bold values are statistically significant.

**P < .01.

^a^ *H*‐statistic for Kruskal‐Wallis test.

^b^ Denotes monaurally obtained aided AzBio sentences in quiet score in the ear to be implanted.

^c^ Chi‐square statistic for chi‐square test.

**Figure 1:**
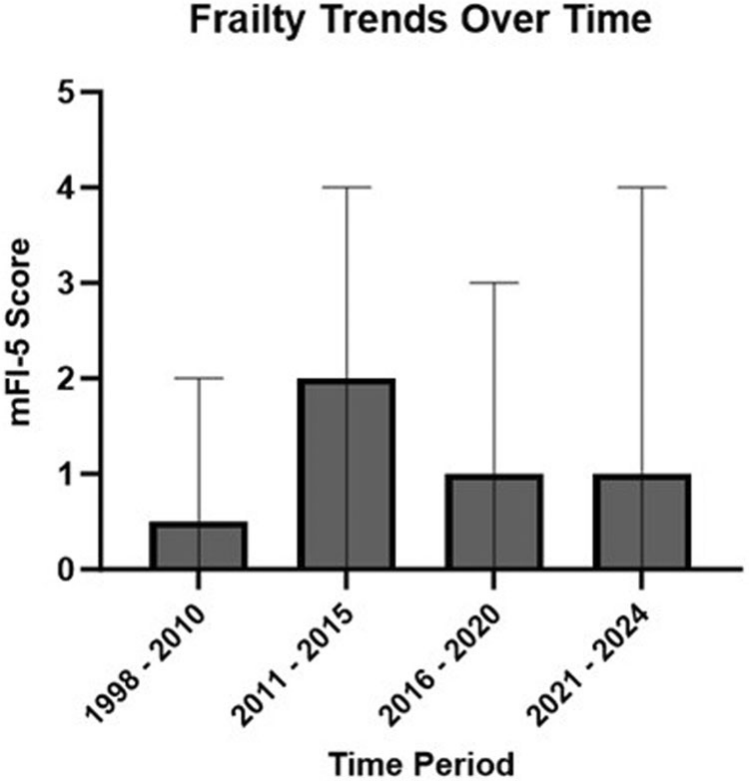
Median and ranges for preoperative frailty (mFI‐5 score) in veteran patients over 25 years of implantation. Frailty scores did not qualitatively explain the increased admission rates over 2016 to 2020.

Admission trends changed over 25 years of offering CI. From 1998 to 2010, all patients (100%) were admitted post‐operatively (4 years, 4 admissions). Rate of admission trended down from 2011 to 2015, with 5 admissions occurring from 24 surgery encounters (20.8%). Twenty‐two (48.9%) patients were admitted from 2016 to 2020 out of 45 encounters; admission rate once again decreased from 2021 to 2024 with 10 (32.3%) patients admitted out of 31 encounters. During 1998 to 2015, only two veterans (7.14%) suffered from postoperative dizziness. Seven (16%) patients experienced postoperative dizziness or vertigo from 2016 to 2020, while 9 (29%) patients experienced postoperative dizziness from 2021 to 2024. Trends in frailty of patients undergoing cochlear implantation over these time periods are depicted in Figure 1. While our system had an uptick in rate of admission from 2016 to 2020, there was not a corresponding increase in patient frailty level during this time. Postoperative dizziness had a significantly higher rate of admission from the PACU (*P* = .005, relative risk 2.25, 95% CI 1.36‐3.42) on Fischer Exact test.

Chi‐square analysis of frailty and admission rate did not reveal a significant difference in risk of admission (*P* = .23) despite that patients with increasing mFI‐5 scores were admitted at higher rates, as in Table 1. Frail patients (mFI‐5 ≥ 2) were admitted (42.9% vs 36.4%, RR 1.15) and experienced postoperative dizziness (20.4% vs 14.5%, RR 1.23) at higher rates than nonfrail and prefrail patients (mFI ≤ 1), though these findings did not reach statistical significance (*P* > .05). Frail patients (mFI‐5 ≥ 2) had a significantly higher rate of death at time of data collection versus nonfrail and prefrail patients (*P* = .03, RR 1.50, 95% CI 1.04‐2.09). Length of admission (LOA) did not differ significantly by mFI‐5 score, but multiple frail patients had prolonged hospitalizations (maximum 8 days), whereas nonfrail and prefrail patients had maximum LOA of 3 and 2 days, respectively.

Table 2 details rate of complications per mFI‐5 score. Surgical complications included vertigo/dizziness (18, 17.3%), pain control issues (3, 2.9%), taste change (2, 1.9%), infection (2, 1.9%), facial nerve stimulation during programming (1, <1%), and receiver‐stimulator migration (1, <1%). Without inclusion of dizziness/vertigo, overall surgical complication rate was 8.6%. One patient had a major surgical complication of infection with resultant implant extrusion, requiring eventual explantation (1, 0.8%). Three patients with dizziness suffered fall events (16.7%, n = 18), of which 1 patient (33.3%) was prefrail (mFI‐5 = 1) and 2 patients (66.6%) were frail (mFI‐5 ≥ 2). Anesthesia complications included cardiac events such as arrhythmias, myocardial infarction, and electrocardiogram changes (ECG) in 5 (4.8%) patients; urinary retention in 4 (3.8%) patients; transient ischemic event and seizure in 2 (1.9%) patients; and allergic reaction in 1 (<1%) patient. Ninety‐day mortality was 0%. Multivariate logistic regression identifying associated patient demographic factors and comorbid diagnoses are shown in Table 3.

**Table 2:** Rates of Surgical and Anesthesia Complications and Common Medical Reasons for Admission per Mfi‐5 Score

mFI‐5 scores					
N (%)	0 Nonfrail	1 Prefrail	2 Frail	3 Frail	4 Frail
Surgical Complications	7/26 (27%)	6/29 (21%)	9/37 (24%)	2/9 (22%)	0/3 (0%)
Anesthesia Complications	1/26 (4%)	3/29 (10%)	6/37 (16%)	1/9 (11%)	1/3 (33%)
Dizziness/Vertigo^a^	4/26 (15%)	4/29 (14%)	8/37 (22%)	2/9 (22%)	
Adm. due to Dizziness & PONV	2	3	6	1	
Falls		1/29 (3%)	2/37 (5%)		
Adm. reason (n)^b^	Cardiac & Pain (1)	Pain, UR (2); Cardiac (1)	TIA & Cardiac (2)	Cardiac (1)	UR (1)

Two (5%) admissions were for non‐medical, social reasons. Reason for admission was unknown for 14 (34%) admissions.

Abbreviations: TIA, transient ischemic attack; UR, urinary retention.

^a^ Not all patients with dizziness/vertigo were admitted; if they felt comfortable discharging and did not have significant PONV (postoperative nausea and vomiting), they were allowed to discharge.

^b^ Common reasons for admission excluding dizziness.

**Table 3:** Multivariate Logistic Regression Analysis Regarding Outcome Variables of Interest and Patient Demographic Factors and Comorbid Diagnoses

Outcome					
Independent variables	Admission OR (95% CI)	Post‐op dizziness OR (95% CI)	Anes. Comp. OR (95% CI)	Surg. Comp.^a^ OR (95% CI)	Vital status OR (95% CI)
Age (years)	1.00 (0.96‐1.05)	0.95 (0.89‐1.01)	1.15* (1.01‐1.30)	0.94 (0.87‐1.01)	1.04 (0.96‐1.12)
mFI‐5	1.51 (0.33‐6.91)	0.37 (0.04‐3.75)	1.93 (0.19‐19.34)	3.39 (0.26‐45.57)	8.14* (1.06‐62.34)
Post‐op Dizziness	4.00* (1.15‐13.86)		0.26 (0.02‐3.96)		0.08 (0.01‐1.14)
Distance (miles)	1.00 (0.99‐1.01)	1.00 (0.99‐1.00)	1.00 (0.99‐1.01)	1.00 (0.99‐1.01)	1.00 (0.99‐1.00)
HTN	0.45 (0.06‐3.57)	8.71 (0.39‐193.70)	0.75 (0.02‐30.04)	0.09 (0.00‐3.44)	0.28 (0.02‐23.97)
CAD	3.71* (1.06‐2.93)	1.60 (0.37‐6.92)	1.23 (0.19‐7.79)	1.94 (0.21‐17.68)	8.59* (1.15‐23.84)
A Fib.	0.93 (0.19‐4.51)	1.69 (0.25‐10.95)	4.01 (0.47‐34.00)	3.14 (0.32‐30.60)	6.63* (1.03‐42.71)
COPD	1.60 (0.22‐11.70)	2.68 (0.19‐37.85)	0.32 (0.02‐5.59)	0.03 (0.00‐4.61)	0.32 (0.02‐4.19)
T2DM	0.83 (0.11‐5.28)	1.36 (0.11‐16.24)	0.78 (0.04‐15.32)	0.20 (0.01‐4.61)	0.04* (0.00‐0.68)
OSA	0.29 (0.07‐1.15)	0.26 (0.04‐1.48)	1.24 (0.15‐10.58)	1.77 (0.25‐12.50)	0.29 (0.03‐1.95)
CKD	0.33 (0.06‐1.70)	0.51 (0.07‐3.63)	1.81 (0.23‐14.32)	7.91 (0.54‐115.86)	1.74 (0.29‐10.40)
CHF	0.94 (0.08‐10.66)	9.54 (0.5‐191.91)	0.29 (0.01‐11.99)	0.22 (0.01‐10.39)	0.16 (0.01‐3.50)

Abbreviations: A Fib., atrial fibrillation; CAD, coronary artery disease; CHF, congestive heart failure; CI, confidence interval; CKD, chronic kidney disease; COPD, chronic obstructive pulmonary disease; HTN, hypertension; OR, odds ratio; OSA, obstructive sleep apnea; T2DM, type 2 diabetes mellitus.

^a^ Denotes surgical complication not including post‐operative dizziness.

*P < .05, **P < .01.

Table 4 demonstrates a multivariate linear regression analysis including patient age and mFI‐5 score. Pre‐implantation monaurally aided AzBio sentence scores in quiet for the ear to be implanted were positively associated with mFI‐5 scores. Interestingly, at the 3‐month post‐CI time point, no such relationship was seen. At 6 months post‐CI, mFI‐5 score was negatively correlated with AzBio sentence scores in quiet. A similar relationship was seen for CNC wordlist in quiet scores, but this did not reach significance. This association, however, was statistically significant and much stronger for both CNC and AzBio sentences in quiet at the 12‐month mark. Age was not a significant predictor of post‐CI WRS outcomes.

**Table 4:** Multivariate Linear Regression Analysis Comparing Pre‐CI And Post‐CI WRS With Patient Age and Preoperative Frailty

Ind. variables	WRS outcomes						
	Pre‐CI	Scores at 3 months		Scores at 6 months		Scores at 12 months	
	AzBio *β* (95% CI)	CNC *β* (95% CI)	AzBio *β* (95% CI)	CNC *β* (95% CI)	AzBio *β* (95% CI)	CNC *β* (95% CI)	AzBio *β* (95% CI)
R‐squared	0.07	0.05	0.03	0.12	0.15	0.36	0.30
Age	−0.03 (−0.45‐0.33)	−0.21 (−1.20‐0.33)	−0.18 (−1.45‐0.51)	−0.23 (−1.11‐0.18)	−0.19 (−1.14‐0.27)	0.07 (−0.56‐0.91)	0.04 (−0.73‐0.94)
mFI‐5	0.27* (0.99‐8.62)	−0.05 (−7.79‐6.01)	0.05 (−7.70‐10.28)	−0.20 (−11.03‐2.44)	−0.31* (−14.25 to −0.18)	−0.62** (−20.24 to −7.12)	−0.56** (−22.2 to −6.6)

Abbreviations: CI, confidence interval; *β*, standardized beta coefficient.

*P < .05, **P < .01.

**Figure 2:**
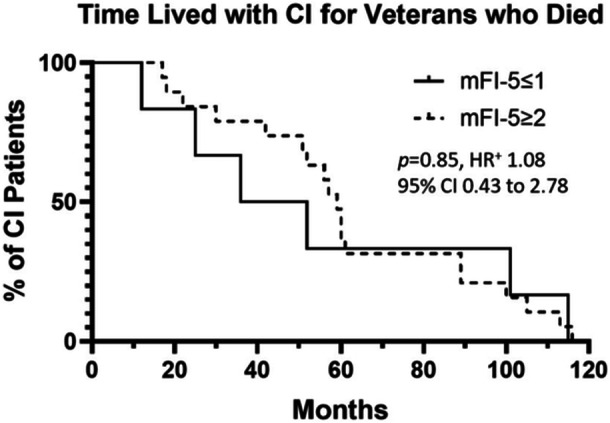
Time lived with CI for patients who had passed at any time during the 25‐year study period. ^+^Hazard ratio abbreviated as HR.

A Kaplan‐Meier analysis was performed among the patients in our study who had died at time of data query by separating patients into frail and prefrail (mFI‐5 ≤ 1) and frail (mFI‐5 ≥ 2) to assess whether frail patients had less years with which to use their implant. Frailty, however, did not impact time lived with CI among patients who had died (Figure 2).

## Discussion

Frailty is known to impact postoperative outcomes;^22^, ^23^ veterans have a higher prevalence of chronic conditions and unique healthcare needs when compared to the general population, which generated our study's question of how frailty in the veteran population interacts with CI outcomes. Our findings indicate that (1) veterans undergoing cochlear implantation seem to have higher rates of frailty than civilian patients when compared to previously published literature, (2) preoperative frailty is not significantly associated with postoperative complications or need for admission, and (3) preoperative frailty has an association with WRS in the first year after implantation, particularly as time advances towards the 1‐year mark.

The use of patient frailty as a preoperative indicator of postoperative outcomes in the field of otolaryngology, in general, has been less compared to other surgical specialties.^27^ Cochlear implantation is a particularly interesting procedure for this metric because of the intersection between older, medically‐complex patients and a purely elective surgery.^13^, ^14^, ^30^ Consequently, several studies have analyzed frailty in CI patients.^13^, ^14^, ^29^, ^31^ Fenton et al demonstrated that frail patients were more likely to have lower pre‐ and post‐implantation WRS and were less likely to become high‐performing CI users within the first 7 months of CI use.^30^ Similarly, any frailty in this study was associated with poorer CI performance. Yuen et al. assessed CI outcomes in terms of WRS and patient‐reported outcome measures (PROMs), showing that, as a whole, patients who were prefrail and frail had similar outcomes to patients with no frailty at 12‐months post‐implantation.^13^ Neither of these studies, however, included information regarding admission and complication rates. Gordon et al. specifically showed that patient frailty and age were not associated with a higher risk of post‐CI complications, but this study did not address admission rate or postoperative dizziness.^14^ Frail patients had lower WRS post‐implantation due to difficulties in accessing post‐operative care when compared to their nonfrail counterparts in Kay‐Rivest et al; their study also showed increasing rates of temporary post‐implantation vertigo among phenotypically frail patients.^31^ Our cohort (46% mFI‐5 score ≥2) is significantly more frail than other cohorts from the available literature (ranging from 10% to 28% mFI‐5 ≥2) that have been assessed with a similar metric,^13^, ^14^, ^29^ which suggests this may be a distinct difference in the veteran CI population compared to the civilian population.

Many physicians who care for patients in a VHA setting have remarked on the unique needs that these patients require from a perioperative standpoint, which specifically encouraged our team to not only characterize our post‐CI WRS, but to also assess admission and complications in our cohort as it relates to preoperative frailty. Totten et al, Tripathi et al, and Petito et al demonstrated that veteran patients have similarly good benefit from CI when compared to civilian patients, but did not include discussion of need for admission and preoperative frailty.^4^, ^33^, ^34^ The high frailty rate of our cohort may be partly responsible for higher rates of admission and perioperative complications. It was reassuring that, while our overall admission rate was high (39.4%), true surgical complication rates were significantly lower (8.6% vs 25.9%) when dizziness/imbalance was excluded. We used a low threshold to include postoperative patient complaints as complications when reviewing documentation to maximize data capture. We specifically chose to report dizziness as a complication to assess if the presence of dizziness was related to frailty and if it had a relationship to post‐operative admission. Admission rate from the PACU and peri‐operative complication rate were not tied to patient frailty, but the frequency of patients reporting symptomatic dizziness after CI increased as mFI‐5 scores increased. This relationship, however, did not reach significance in our cohort.

The present study supports the notion that frailty negatively influences CI benefit, which has previously been suggested. Preoperatively, AzBio sentence list scores in quiet did not correlate negatively with patient frailty. Throughout the first year after implantation, the association between frailty scores and WRS evolved from no clear association at 3‐month to a strong, negative relationship at 12‐month for both AzBio and CNC scores. We hypothesize this finding may derive from decreased time available for auditory rehabilitation among patients with numerous chronic conditions; these patients, with their families and caregivers, may need to prioritize other aspects of their healthcare. Cognition, cochlear health, and age can be significant confounding variables as well, though based on these factors, we would have expected to see similar relationships evident from preoperative to all postoperative timepoints.

Our study is retrospective and carries bias related to this method of research. Electronic medical record review may cause omission of chronic conditions and complications due to incomplete documentation, and in general, the availability of data for patients traveling from other VA systems. The medical record did not always identify the reason for post‐operative admission; anecdotally, admissions can occur due to transportation availability or social reasons. Though we do not have a full dataset of PROMs to report for this cohort, it is very likely that frail patients experience significant benefit from their implants despite having lower WRS.^13^ Our study does not include datalogging information, which we plan to investigate sequentially. Age and cognitive ability are noteworthy as possible confounding factors in our study design. We clearly demonstrate pre‐operative frailty as a significant associated variable when age is appropriately accounted for, but we were unable to include cognition, duration of hearing loss, and datalogging due to a small cohort and insufficient data, which precludes meaningful multivariate analysis with these variables. Lastly, we were not able to include long‐term data (over 1 year after implantation) for our cohort to determine if frail patients have similar results after the study period.

## Conclusion

Veterans undergoing CI have higher rates of frailty when compared to the general population, which may affect perioperative complication and admission rates for this population. Within our cohort, pre‐operative frailty did not significantly predict the need for admission from the PACU or postoperative dizziness and imbalance, but speech recognition outcomes at 1‐year after implantation were worse for frail patients. Frail veterans with severe hearing loss still experience audiologic benefit from cochlear implantation, but future investigations are necessary to assess and address worse speech outcomes for these patients. Furthermore, these patients may benefit from CI‐specific enhanced recovery protocols to minimize complications and anticipate the need for admission/observation.

## Author Contributions

**Kaitlyn A. Brooks**, study design, data collection, analysis, presentation, manuscript writing; **Benjamin D. Lovin**, study design, data collection, manuscript writing; **Alex D. Sweeney**, study design, manuscript writing; **Angela S. Peng**, study design, analysis, manuscript writing, presentation; **Nathan R. Lindquist**, study design, data collection, analysis, presentation, manuscript writing.

## Disclosures

### Competing interests

Alex D. Sweeney: Consultant for Cochlear Americas, research funding from Cochlear Americas. Past consultant/surgical advisory board for Advanced Bionics, Oticon Medical, MED‐EL. The remaining authors testify no financial relationships to disclose or conflicts of interest pertaining to this work.

### Funding source

None.
